# Liver segmentation from CT images using a sparse priori statistical shape model (SP-SSM)

**DOI:** 10.1371/journal.pone.0185249

**Published:** 2017-10-05

**Authors:** Xuehu Wang, Yongchang Zheng, Lan Gan, Xuan Wang, Xinting Sang, Xiangfeng Kong, Jie Zhao

**Affiliations:** 1 School of Electronic and Information Engineering, Hebei University, Baoding, China; 2 Key Laboratory of Digital Medical Engineering of Hebei Province, Baoding, China; 3 Department of Liver Surgery, Peking Union Medical College Hospital, Chinese Academy of Medical Sciences and Peking Union Medical College, Beijing, China; 4 School of Information Engineering, East China Jiaotong University, Nanchang, China; Chongqing University, CHINA

## Abstract

This study proposes a new liver segmentation method based on a sparse a priori statistical shape model (SP-SSM). First, mark points are selected in the liver a priori model and the original image. Then, the a priori shape and its mark points are used to obtain a dictionary for the liver boundary information. Second, the sparse coefficient is calculated based on the correspondence between mark points in the original image and those in the a priori model, and then the sparse statistical model is established by combining the sparse coefficients and the dictionary. Finally, the intensity energy and boundary energy models are built based on the intensity information and the specific boundary information of the original image. Then, the sparse matching constraint model is established based on the sparse coding theory. These models jointly drive the iterative deformation of the sparse statistical model to approximate and accurately extract the liver boundaries. This method can solve the problems of deformation model initialization and a priori method accuracy using the sparse dictionary. The SP-SSM can achieve a mean overlap error of 4.8% and a mean volume difference of 1.8%, whereas the average symmetric surface distance and the root mean square symmetric surface distance can reach 0.8 mm and 1.4 mm, respectively.

## Introduction

The liver is the largest digestive gland and detoxification organ in the human body, and this vital organ also produces bile. Therefore, the integration of multiple functions makes the liver one of the major organs most prone to tumors. In recent years, liver cancer has become the second most common cause of cancer deaths worldwide. Therefore, the prevention and treatment of liver diseases have been a focus worldwide. Computed tomography (CT) imaging can be used to acquire high-resolution hepatic anatomical structures. If hepatic lesions occur, they can be found on the CT image by their characteristic inhomogeneous intensity distribution and unsmooth edges. Therefore, CT imaging has represented of the most important imaging techniques for the diagnosis and treatment of clinical liver diseases. Live segmentation can help identify the liver contour and lesion structures from other tissues; thus, it is very useful in the clinical application of liver function evaluations, tumor identification and surgical treatment. However, the liver is adjacent to other organs, such as the spleen, stomach and intestines, and the intensity features of these organs present very small differences. Furthermore, the liver has strong individual differences, and its structure and spatial position are easily subject to changes under external forces. Thus far, accurate segmentation and detection of the liver contour in a CT image remains the most challenging task worldwide.

In recent years, researchers have proposed many liver segmentation methods based on CT images. Among them, the method based on statistical models and probabilistic graphical models can effectively leverage the real-time features of the liver. In this case, the liver image can be effectively segmented based on imaging parameters and the pathogeny structure. However, this method requires an extremely large sample size, which greatly decreases the segmentation efficiency. When sparse coding is applied, the training samples for the statistical model and probabilistic graphical model can be represented sparsely, which helps remove redundant information, reduce the calculation amount, and improve segmentation efficiency. [1–3] Wright et al. [4] used this method to create a dictionary in the target area of the training image and partition the image into several image blocks of the same size to establish the test set. Accordingly, the liver’s boundaries can be determined based on the matching degree of the test set and the dictionary. This method effectively reduces the redundancy information in the training image and improves the computational efficiency by specifying a sparse coefficient corresponding to the dictionary. Ptucha et al. [5] proposed a liver segmentation method based on the sparse dictionary. The Singular Value Decomposition (K-SVD) algorithm [6–10] is used to train the dictionary and classifier, and the liver boundaries are determined based on the matching degree of the image and the dictionary. Subsequently, the liver can be segmented with less computational complexity. Cao et al. [11] uses the gold standard image that is segmented manually by experts as a priori information. In this method, the sparse coding and dictionary are combined to obtain a sparse representation matrix for different categories. Then, the image reconstructing algorithm is applied to obtain the final segmentation result. However, if the intensity information contained in the a priori image is not aligned with that of the original image, the segmentation accuracy will be compromised. Liao, Tong, et al. [12–13] replaced voxel imaging information with local image block features, which moderately improved the segmentation accuracy of the low-resolution area. Guo et al. [14] introduced a sparse optimization model based on a priori information. In this method, the liver surface is partitioned into small sub-regions, the K-SVD algorithm is applied to build the shape dictionary, and the variation trend of the deformable model is constrained via sparse shape information. Saito et al. [15] integrated sparse representation with a priori shape information and applied a hierarchical analysis to partition the deformable model into small sub-regions. Subsequently, the sparse codes and dictionary are constructed independently based on the local shape model, thereby reconstructing the image for liver segmentation.

The major deficiency in the abovementioned methods occurs when establishing the sparse model. To build the sparse matrix and dictionary, similar image blocks must be selected from the original image. However, the similarity matching of image blocks presents limited accuracy. In addition, the Discrete Cosine Transform (DCT) [16] dictionary is typically used in these methods to obtain the similarity measure of images, which leads to low matching accuracy. The segmentation result may represent different holes and overlaps, thereby compromising the accuracy of the segmentation. Furthermore, the accuracy and efficiency of liver segmentation are directly related to the size of image blocks. In addition, the method based on a statistical model requires the model after registration as the initial boundary and the selected image blocks around the initial boundary as test sample sets because large deformation regions of the liver cannot be accurately segmented. In other areas of medical image segmentation, researchers have proposed high-performance algorithms [17–20] that can represent a good reference for this paper.

To solve these problems, this paper proposes a new method called the Sparse A Priori Statistical Shape Model (SP-SSM), which is based on grayscale images to be segmented and the specificity of the border, and these features are used to build the energy model. The method is then combined with the query and dictionary matching gray features to build a sparse constraint-driven iterative sparse statistical shape model deformation that approaches the liver border to form an accurate extraction of the liver boundary.

## Methods

**Fig 1:**
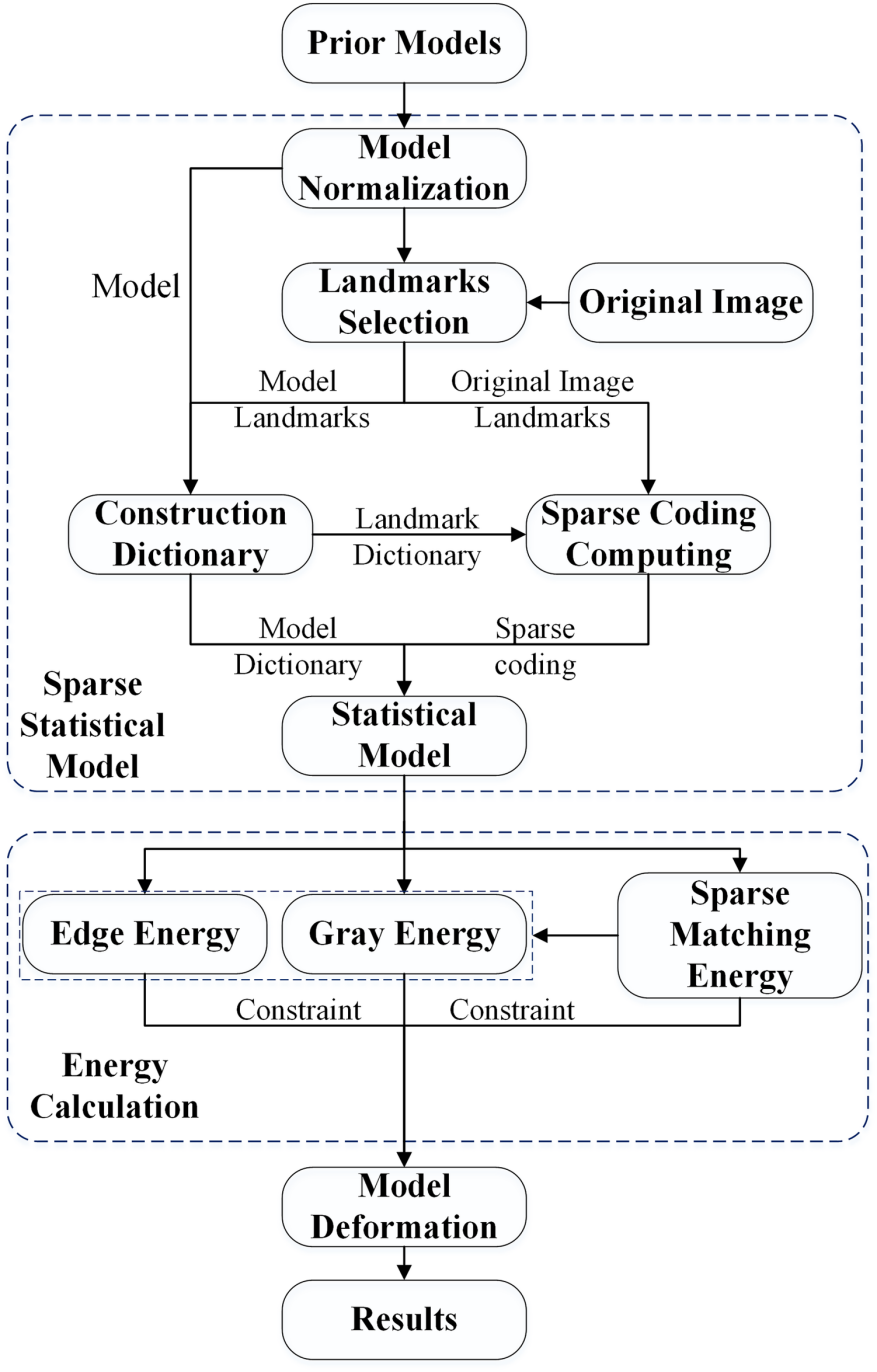
Flowchart of the proposed algorithm.

The statistical shape model based on sparse coding proposed in this study includes eight key steps: (1) use the generalized Procrustes analysis (GPA) to normalize the a priori shape models, enable each group of a priori models to have the same number of vertexes, and ensure that the vertexes of each group correspond to those of the other a priori models; (2) choose the corresponding vertexes from all a priori models to be used as the mark points; (3) create the inquiry dictionary using the a priori shape models and their corresponding mark points; (4) manually select the mark points corresponding to the a priori models from the original image and use the dictionary for mark points to calculate the sparse codes; (5) use the sparse codes for the mark points and the dictionary for a priori models to build the sparse statistical shape model; (6) obtain the corresponding intensity values of the vertexes of the sparse statistical shape model on the original image and calculate the specific boundaries of the original images to build the boundary energy and the intensity energy; (7) choose image blocks at the gold standard liver boundaries as the training sets to obtain the inquiry dictionary and the sparse codes and construct the sparse matching constraint model based on the dictionary and the intensity information of the original image; and (8) deform the statistical shape model driven by the intensity energy, boundary energy and sparse matching constraints until the segmentation is complete. Fig 1 shows the flowchart of the proposed algorithm.

### Sparse statistical shape model

**Fig 2:**
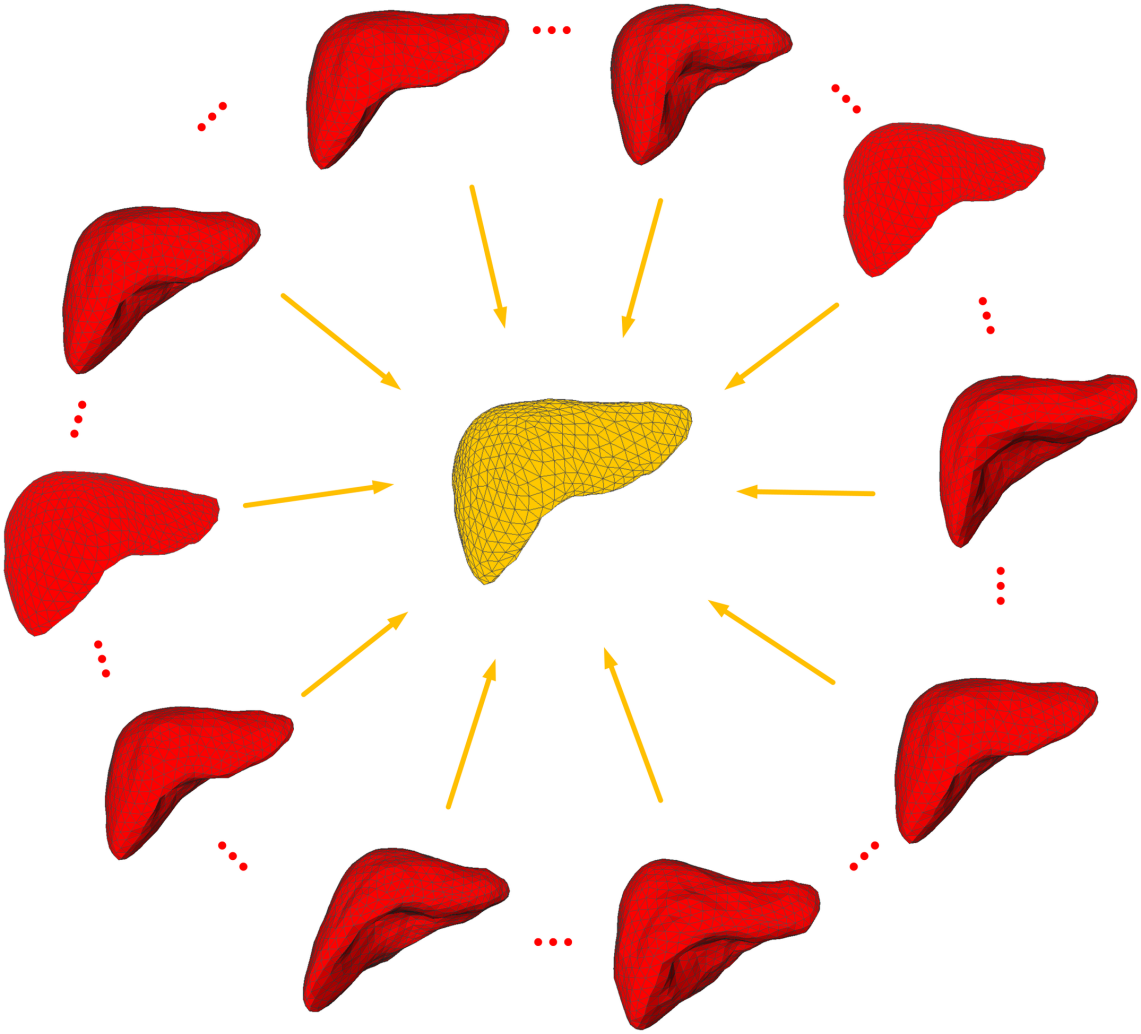
Illustration of how to build a sparse statistical shape model.

The sparse statistical shape model uses multiple known shapes to train a model that can effectively describe the distribution of various points. The main concept underlying the model’s construction is shown in Fig 2. The traditional method of building a statistical shape model is to manually select a large number of mark points from the data and sort the mark points to form a model that can roughly describe the target shape. Then, multiple shape models are selected via multiple training images, and those images are registered. Finally, the Principal Component Analysis (PCA) method is used to reduce their dimensions and build a model that can describe the distribution of the target vertexes. Because the variation of liver shapes is complex, the statistical model built using a probability distribution may have significant errors. Additionally, the process of reducing model dimensions using the PCA method may lead to the loss of the important local structural information on the liver's sharp corners, grooved regions, and other tricky regions. To address these problems, this study proposes a statistical shape model based on sparse coding. The main concepts underlying the method are as follows: (1) normalize the a priori shape models of the liver, *i*.*e*., align them to the same shape space; (2) choose orderly mark points that can effectively describe the liver's features from the aligned a priori models; (3) create dictionaries *D*_*S*_ and *D*_*L*_ using the a priori models and mark points, respectively; (4) choose the orderly mark points that present the same approximate positions on the original image to create the test set *Y*_*L*_; (5) use the test set *Y*_*L*_ and the mark point dictionary *D*_*L*_ to calculate the corresponding sparse coefficient *X*_*L*_; (6) apply *X*_*L*_ to *D*_*S*_ to build the a priori statistical model *Y*_*S*_. The modeling algorithm mainly involves three parts: a priori model normalization, mark point selection, and model building.

#### A priori model normalization

The a priori shapes of the livers have individual differences in location, size, and direction. Before building a sparse statistical model, we must align the various models to reduce the differences. Traditionally, a Procrustes analysis is used to align all the models, which allows for various a priori shapes to closely approximate each other by translating, rotating and enlarging them without changing their original shapes. This method requires that the vertexes of the models correspond to each other. However, the a priori shape models of different livers have different numbers of vertexes (*i*.*e*., n is different). Therefore, this study uses the gold standard image from MICCAI 2007 (http://www.sliver07.org/index.php) to serve as the liver's a priori model and applies the down-sampling method to obtain the a priori models of the same number of vertexes and facets. Subsequently, a Procrustes Analysis is implemented to normalize the models to ensure that the vertexes of each group of a priori models correspond to each other. If the a priori shape is defined as *S*_*i*_ and the average shape is defined as $\bar{S}$, then the Procrustes distance between the two groups of a priori models *S*_1_ and *S*_2_ can be described as follows:

$$P_{d}^{2}=\sum_{j=1}^{n}\left\{{\left(S_{1}-S_{2}\right)}^{2}\right\}=\sum_{j=1}^{n}\left\{{\left(x_{j1}-x_{j2}\right)}^{2}+{\left(y_{j1}-y_{j2}\right)}^{2}\right\}$$ (1)

where *x*_*j*1_ and *y*_*j*1_ indicate the vertex coordinates in a priori model *S*_1_ and *x*_*j*2_ and *y*_*j*2_ represent the vertex coordinates in a priori model *S*_2_.

The barycentric coordinates of each group of a priori shape models are calculated as follows:

$$\left(\bar{x},\bar{y}\right)=\left(\frac{1}{n}\sum_{j=1}^{n}x_{j},\frac{1}{n}\sum_{j=1}^{n}y_{j}\right)$$ (2)

The size of each group of a priori shapes is defined by using the Euclidean/Frobenius norm [21] as follows:

$$R\left(n\right)=\sqrt{\sum_{j=1}^{n}\left\{{\left(x_{j}-\bar{x}\right)}^{2}+{\left(y_{j}-\bar{y}\right)}^{2}\right\}}$$ (3)

The aligning process of the two groups of a priori shape models in the training set is shown below:

1. Define the transformation vectors of the two groups *S*_1_ and as *d*_1_ and *d*_2_, respectively:
2. *x*, *y*, *s* and *θ* represent the translating, magnifying and rotating operations in the horizontal and vertical directions, respectively, based on which the parametric equation can be defined as: t = (*x*, *y*, *s*, *θ*);
3. Calculate the barycentric coordinates of the two a priori shape models;
4. Magnify each a priori shape model so that they have the same size;
5. Align the barycenters of the two models;
6. Use the SVD method to rotate and align the two models:
7. Calculate the SVD and *R*_*T*_ of $d_{1}^{T}d_{2}$;
8. Generate the result.

Whereas the aligning process of all a priori shape models is shown below:

1. Translate the barycenter of each a priori shape model to the origin of the coordinates;
2. Use the first a priori shape model as the initial estimation of the average model;
3. Rotate, magnify and translate the other a priori shape models to align them with the current average model;
4. Recalculate the average model of all a priori shape models after they are aligned: $\bar{S}=\frac{1}{k}\sum_{i=1}^{k}S_{i}$;
5. Estimate the difference between the current average model and the previous average model. If they are not converged (the error is smaller than the threshold value *ε*), skip to Step 3; if they are converged, then the process is complete.

Among them, the rotation matrix *R*_*T*_ is defined as follows:

$$\begin{array}{ccc} R_{T} & = & R_{x}R_{y}R_{z}\\ & = & \left[\begin{array}{ccc} 1 & 0 & 0\\ 0 & \text{cos}\left(\phi \right) & -\text{sin}\left(\phi \right)\\ 0 & \text{sin}\left(\phi \right) & \text{cos}\left(\phi \right) \end{array}\right]\left[\begin{array}{ccc} \text{cos}\left(\psi \right) & 0 & -\text{sin}\left(\psi \right)\\ 0 & 1 & 0\\ \text{sin}\left(\psi \right) & 0 & \text{cos}\left(\psi \right) \end{array}\right]\left[\begin{array}{ccc} \text{cos}\left(\varphi \right) & -\text{sin}\left(\varphi \right) & 0\\ \text{sin}\left(\varphi \right) & \text{cos}\left(\varphi \right) & 0\\ 0 & 0 & 1 \end{array}\right] \end{array}$$ (4)

#### Mark point selection

**Fig 3:**
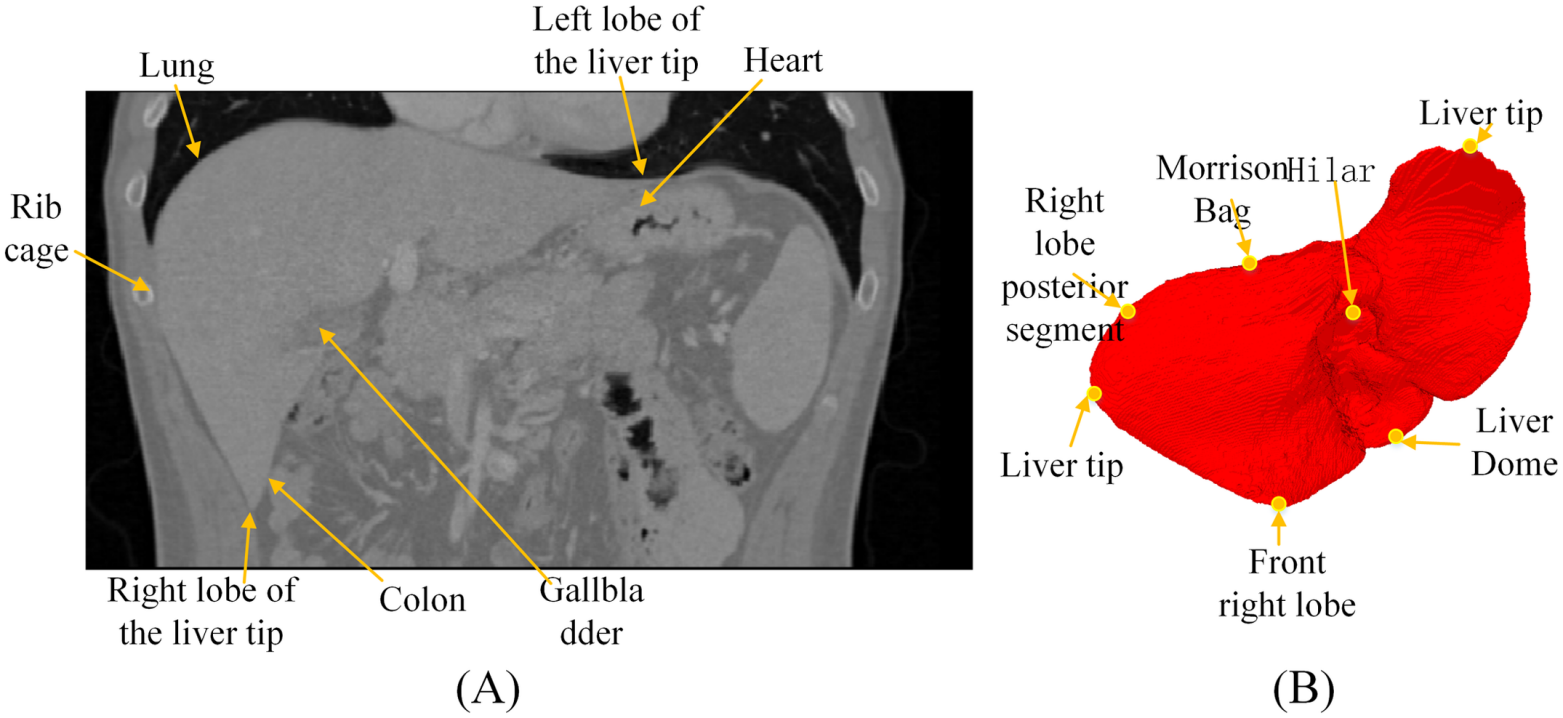
Illustration of mark point selection. (A) Illustrates the position in relation to other tissues; and (B) represents different points of the liver.

After Procrustes alignment, we can obtain the distribution of the a priori shape models and the statistical model that can correctly describe the distribution of the vertexes. Traditionally, a PCA is used for the statistical analysis to determine the weight of the probability distribution by calculating the principal components. For liver models, the sharp corners and grooved regions of different groups of models cannot completely overlap, and they can only become principal components in regions that are relatively smooth. Therefore, the statistical shape models built using this method often may lose information of local features. Thus, in this study, we manually choose mark points for the sharp corners and grooved regions (with a high curvature point or angular point) or regions closely connected with other tissues. In total, we select seven groups of mark points, including Hepatic Dome, Right Lobe Anterior Segment, Right Lobe Tip, Right Lobe Posterior Segment, Morrison Pouch, Porta Hepatis, and Left Lobe Lateral Segment, as shown in Fig 3.

#### Model building

After alignment, the model dictionary *D*_*M*_ = [*d*_*M*1_, *d*_*M*2_, ⋯, *d*_*Mk*_] ∈ *R*^*n*×*k*^ and mark point dictionary *D*_*L*_ = [*d*_*L*1_, *d*_*L*2_, ⋯, *d*_*Lk*_] ∈ *R*^*n*×*k*^ can be built for the model and its corresponding mark point, respectively, where *k* refers to the total number of a priori shape models in the training set, *d*_*i*_ ∈ *R*^*n*^ refers to the vector transferred by a priori models, *n* refers to the number of vertexes following the down-sampling of a priori models, and *y* ∈ *R*^*n*^ refers to the vector transferred by the newly input model. Assuming the newly input models *y* in every unit can be expressed by *d*_*i*_,*i* = 1, 2, 3, ⋯, *k* in the training set in a weighted linear manner, then *x* = {*x*_1_, *x*_2_, ⋯ *x*_*k*_}^*T*^ ∈ *R*^*k*^ should be defined as a weighting factor or coefficient (sparse coding). Then, the value of *x* can be calculated by the following formula:

$$\underset{x,\beta }{\text{arg}\;\text{min}}\left\{{\left\|T\left(y,\beta \right)-Dx\right\|}_{2}^{2}\right\}$$ (5)

where *T*(y, *β*) is a transfer function that can transfer newly input models *y* into average model data space and *β* is a transfer parameter. *x* and *β* can also be calculated by the preceding formula.

**Fig 4:**
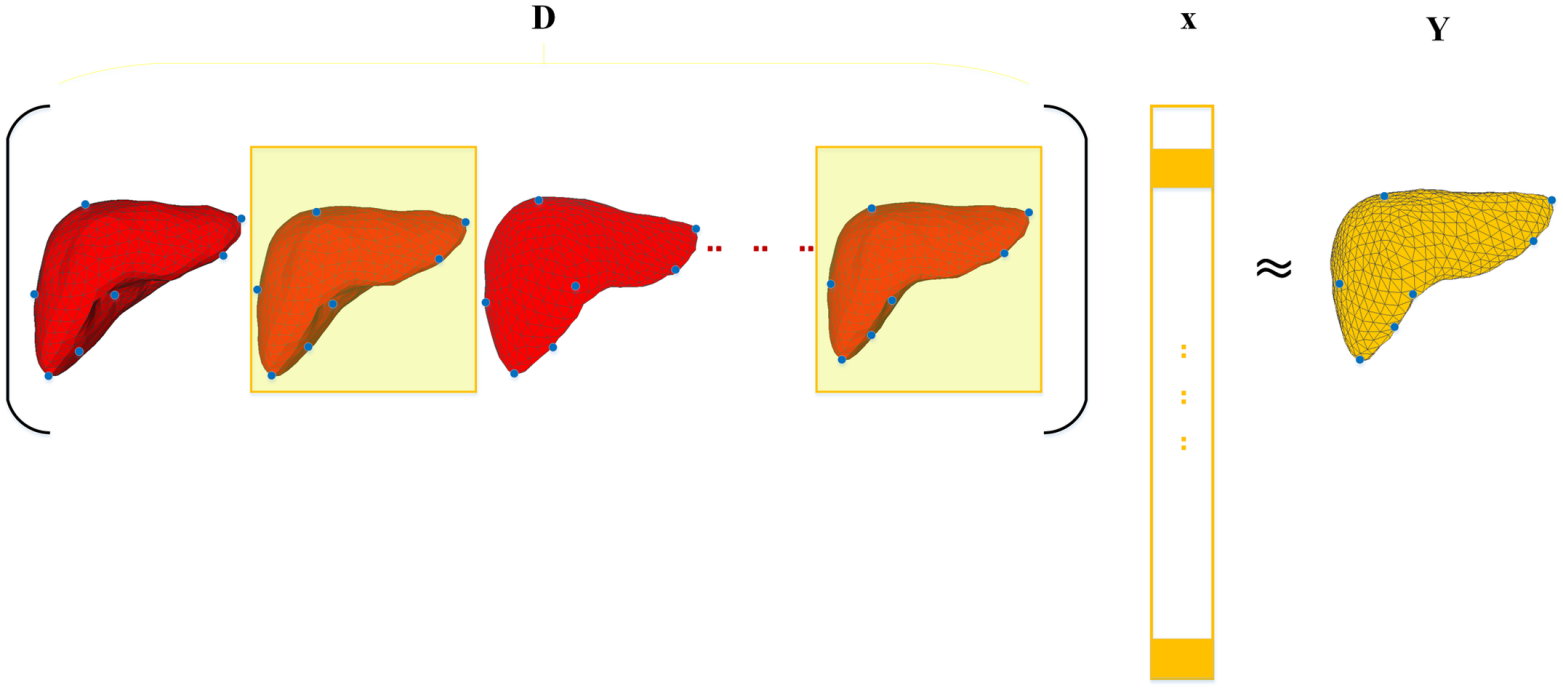
Sketch map for dictionary building.

Fig 4 shows the sketch map of dictionary building and the newly input models, where Y is expressed as *Dx*. When Formula (5) is used to solve *x* and *β*, if the number of models is greater than model length (*k* > *n*), then the formula does not have a unique solution and a bound term is required to control weighting coefficient *x*. In this case, Formula (5) can be transformed as follows:

$$\underset{x,\beta }{\text{arg}\;\text{min}}\left\{{\left\|T\left(y,\beta \right)-Dx\right\|}_{2}^{2}\right\},s.t.{\left\|x\right\|}_{0}\leq k_{1}$$ (6)

where ‖*‖_0_ refers to the zero norm of the vector and *k*_1_ refers to the pre-set sparseness. Then, it can be ensured that the nonzero term in coefficient *x* is less than *k*_1_. The value of *k*_1_ will be discussed in the experiment.

When the input model contains non-Gaussian noise or major errors, such as image masking and pixel loss, the sparse vector *e* ∈ *R*^*n*^ can be defined and used to indicate the model's calculation error. In this case, Formula (6) can be further transformed as follows:

$$\underset{x,e,\beta }{\text{arg}\;\text{min}}\left\{{\left\|T\left(y,\beta \right)-Dx-e\right\|}_{2}^{2}\right\},s.t.{\left\|x\right\|}_{0}\leq k_{1},{\left\|e\right\|}_{0}\leq k_{2}$$ (7)

where *k*_2_ refers to the sparseness of *e*. If errors, such as image masking, are not observed in the input image, then the value of *e* is zero.

Formula (7) can be transformed into the 1-norm for the solution, and the transformed formula is as follows [22]:

$$\underset{x,e,\beta }{\text{arg}\;\text{min}}\left\{{\left\|T\left(y,\beta \right)-Dx-e\right\|}_{2}^{2}+\lambda_{1}{\left\|x\right\|}_{1}+\lambda_{2}{\left\|e\right\|}_{1}\right\}$$ (8)

where *λ*_1_ and *λ*_2_ indicate the weighting coefficients of *x* and *e*, respectively. If *λ*_2_ has a large value, then *e* is zero. If both *λ*_1_ and *λ*_2_ have a large value, then *e* is zero and *x* has only a nonzero term. Formula (8) can be optimized through the K-SVD algorithm.

### Energy

**Fig 5:**
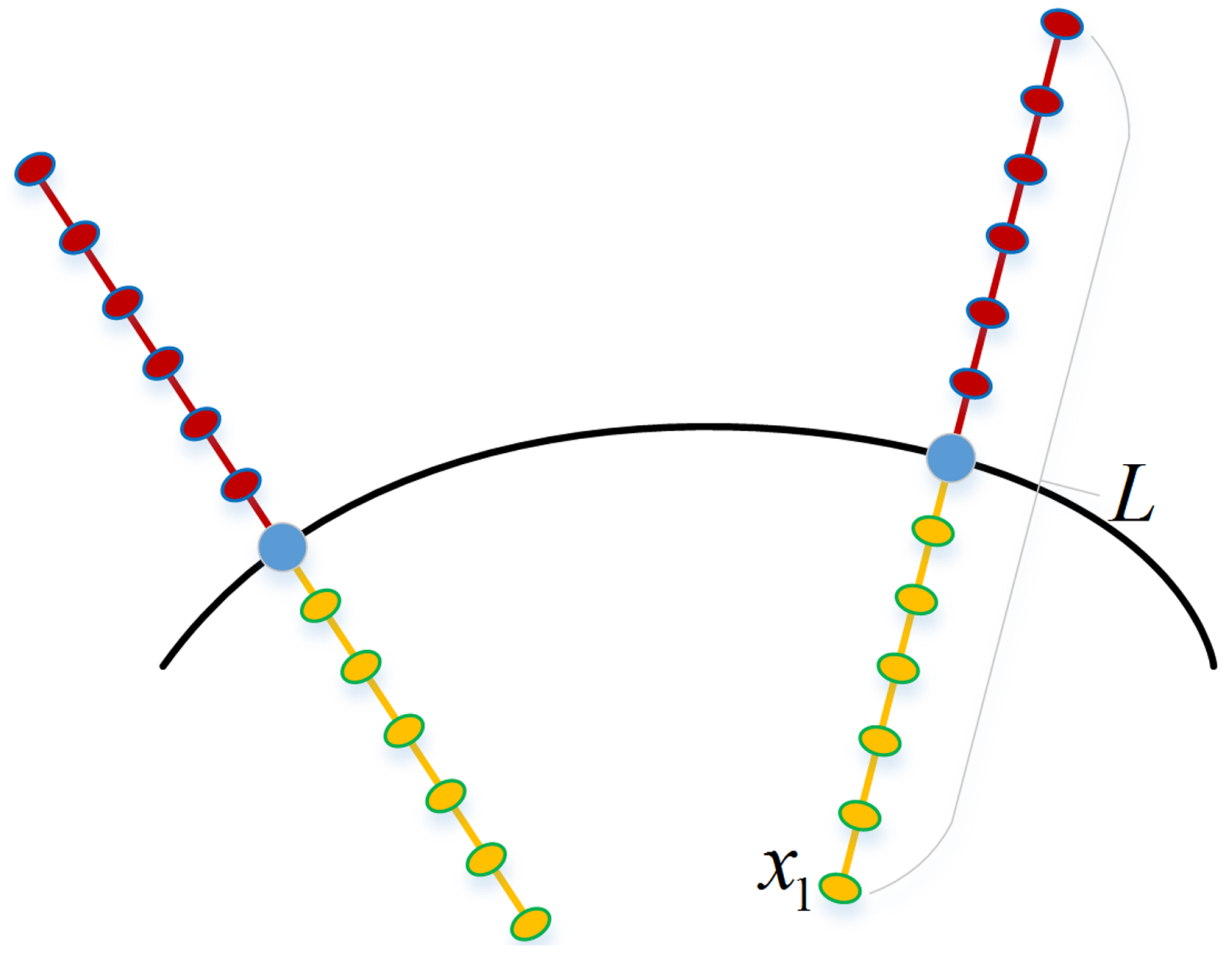
Sketch map for feature selection.

By building sparse statistical shape models, a series of statistical shape expressions of original images are obtained. The sparse statistical shape models are overlaid on the original image *I*_*test*_, and the corresponding intensity information is obtained for the vertex $V={\left\{v_{i}\in R^{3}\right\}}_{i=1}^{n}$. With the coordinate of every vertex in the model as the center, pixels are selected evenly along the normal vector and the intensity level of pixels is defined as *p*_*i*_ and the length is defined as *L* as shown in Fig 5. The coordinate of every pixel can be calculated using the following formula:

$$x_{i}=x+\left[\left(i-1\right)/\left(m-1\right)-1/2\right]\cdot L\cdot u,\overset{}{\underset{}{}}s.t.\overset{}{\underset{}{}}i=1,2,\cdots ,m$$ (9)

where *x* refers to the boundary point, *m* refers to the number of points obtained evenly at the boundary point along the normal vector, and *u* refers to the distance between pixels.

To make the statistical model converge to the real boundary of the liver, this paper proposes the constraints on intensity energy, boundary energy, and sparse matching energy. The energy function can be calculated by the following formula:

$$F_{external}=\omega_{external}\left(E^{edge}\left(x\right)+E^{region}\left(x\right)\right),\overset{}{\underset{}{}}s.t.\overset{}{\underset{}{}}x\in X$$ (10)

where *E*^*region*^(*x*) refers to the intensity energy of a vertex in the statistical shape models, *E*^*edge*^(*x*) refers to the boundary energy of the vertex, and *ω*_*external*_ is the weighting coefficient of the energy under sparse matching constraints. The specific building method is as follows:

#### Intensity energy building

Intensity energy *E*^*region*^(*x*) is estimated based on the intensity level histogram. Intensity energy can be used to roughly separate the liver and its surrounding tissues. This paper adopts an intensity level histogram fitting to the Weighted Gaussian Mixture Model (WGMM) with expectation maximization to obtain five Gaussian distributions *P*_*i*_(*i* = 1, 2, ⋯, 5). We then determine the weight *ω*_*i*_, mean value *μ*_*i*_, mean square error *σ*_*i*_ and peak height *h*_*i*_ = *ω*_*i*_/*σ*_*i*_.

The intensity average *G*_*m*_ of liver tissues is calculated as follows:

$$G_{m}=\left\{\mu_{m}|h_{m}=\text{max}\left(h_{i}\right)\right\},\overset{}{\underset{}{}}s.t.\overset{}{\underset{}{}}i\in \left(1,2,\cdots ,5\right)$$ (11)

The liver intensity range is [*G*_*L*_, *G*_*U*_], where *G*_*L*_ = *μ*_*m*_ − 1.5*σ*_*m*_ and *G*_*U*_ = *μ*_*m*_ + 1.5*σ*_*m*_. For every vertex *x*_*i*_(*i* = 1, 2, ⋯, *n*), *E*^*region*^(*x*) can be calculated based on the procedures as follows:

1. Initialization: *i* = 1;
2. If *I*(*x*_*i*_) ∈ [*G*_*L*_, *G*_*U*_] and *I*(*x*_*i+*1_) ∈ [*G*_*L*_, *G*_*U*_], then *E*^*region*^(*x*) = *x*_*i*_-*x*_*i*−1_;
3. If *I*(*x*_*i*_) ∉ [*G*_*L*_, *G*_*U*_], then *E*^*region*^(*x*) = 0;
4. End.

#### Boundary energy building

**Fig 6:**
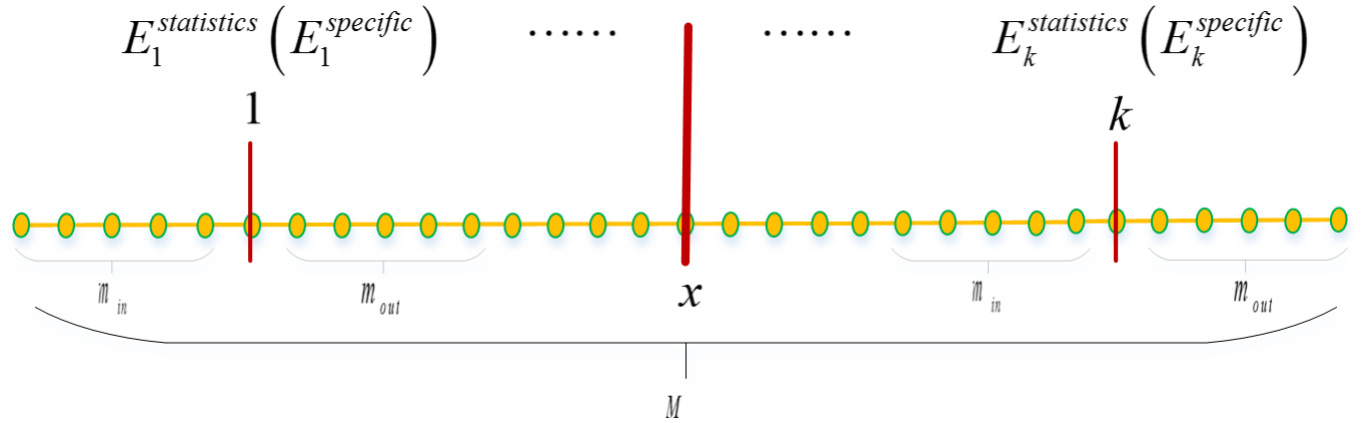
Sketch map for energy function calculation.

We build boundary energy *E*^*edge*^(*x*) according to the edge information indicated by the original data. We define a vertex in the sparse statistical shape models as *v*_*i*_ ∈ *R*, *i* = 1, 2, ⋯, *m*, where R refers to the sparse statistical shape models and *x*_*i*_ is the physical coordinate of *v*_*i*_. We also define the unit normal vector of the vertex as *u*. We then select the intensity value corresponding to M pixels of the normal vector as shown in Fig 6, where *k* = *M* − *m* + 1, *m* = *m*_*in*_ + *m*_*out*_ + 1, and *m*_*in*_ = *m*_*out*_ = 5.

Moreover, the displacement energy function of the edge information can be calculated by the following formula:

$$E^{edge}\left(x\right)=\underset{i}{\text{max}}\left\{\alpha E_{statistics}\left(x_{i}\right)+\beta E_{specific}\left(x_{i}\right)\right\},\overset{}{\underset{}{}}s.t.\overset{}{\underset{}{}}\alpha +\beta =1$$ (12)

where *E*_*statistics*_(*x*_*i*_) and *E*_*specific*_(*x*_*i*_) refer to the statistical feature energy and the specific feature energy of the vertexes, respectively, and they are calculated randomly [23]. For every vertex *v*, *E*_*statistics*_(*x*_*i*_) and *E*_*specific*_(*x*_*i*_) are both the vectors of 1 × *k*.

To solve Formula (12), the statistical feature energy *E*_*statistics*_(*x*_*i*_) is calculated. We then select the shape model Φ and image *I*_*training*_ corresponding to the training set. We also select the intensity feature of every vertex of the shape model based on Formula (10), and as shown in Fig 6, *m*_*in*_ = *m*_*out*_ = 5. We define *z* as the number of shape models for the training set and calculate *μ*_*ij*_ and *σ*_*ij*_(*i* = 1, 2, ⋯, *m*; *j* = 1, 2, ⋯, *n*) for the mark points corresponding to the training set by the following formulas:

$$\mu_{ij}=\frac{1}{z}\sum_{k=1}^{z}p_{ijk}\overset{}{\underset{}{}}\;s.t.\overset{}{\underset{}{}}i=1,2,\cdots ,m;j=1,2,\cdots ,n$$ (13)

$$\sigma_{ij}=\sqrt{\frac{1}{z-1}{\sum_{k=1}^{z}\left(p_{ijk}-\mu_{ij}\right)}^{2}}\overset{}{\underset{}{}}\;s.t.\overset{}{\underset{}{}}i=1,2,\cdots ,m;j=1,2,\cdots ,n$$ (14)

For every vertex, we calculate the boundary probability *P*_*k*_ of 1, 2, ⋯, *n*. Weighted with a Gaussian kernel function *K*(‖*j* − *j*_*middle*_‖), *P*_*k*_ can be calculated by the following formula:

$$P_{k}=\sum_{j=k}^{k+n-1}K\left(\left\|j-j_{middle}\right\|\right)\frac{1}{\sqrt{2\pi }\sigma_{i\left(j-k+1\right)}}e^{\frac{-{\left(p_{j}-\mu_{i\left(j-k+1\right)}\right)}^{2}}{2\sigma_{i\left(j-k+1\right)}^{2}}},\overset{}{\underset{}{}}\;s.t.\overset{}{\underset{}{}}i=1,2,\cdots m$$ (15)

where *j*_*middle*_ = *k* + *m*_*in*_. The statistical feature energy *E*_*statistics*_(*x*_*i*_) is calculated upon the normalization of *P*_1_, *P*_2_, ⋯ *P*_*k*_.

Second, we calculate the specific feature energy *E*_*specific*_(*x*_*i*_). The outer/inner means difference and the outer/inner regularity difference of the intensity information surrounding the liver tissues in the CT image are regarded as specific information for the algorithm in this paper, where the absolute value of the outer/inner means difference is calculated as follows:

$$f^{1}=\left|\bar{p_{in}}-\bar{p_{out}}\right|$$ (16)

In the preceding formula,

$$\bar{p_{in}}=\frac{1}{m_{in}}\sum_{t=1}^{m_{in}}K\left(\left\|t-t_{middle}\right\|\right)p_{t}$$ (17)

$$\bar{p_{out}}=\frac{1}{m_{out}}\sum_{t=1}^{m_{out}}K\left(\left\|t-t_{middle}\right\|\right)p_{t}$$ (18)

$$t_{middle}=m_{in}+1$$ (19)

The outer/inner means difference is calculated as follows:

f2=σout−σin (20)

where

$$\sigma_{in}=\sqrt{\frac{1}{m_{in}-1}{\sum_{t=1}^{m_{in}}\left(p_{t}-\bar{p_{in}}\right)}^{2}}$$ (21)

$$\sigma_{out}=\sqrt{\frac{1}{m_{out}-1}{\sum_{t=1}^{m_{out}}\left(p_{t}-\bar{p_{out}}\right)}^{2}}$$ (22)

We calculate the $f_{t}^{1}$ and $f_{t}^{2}$ of *t* = 1, 2, ⋯, *k* and then conduct normalization and linear additivity for the values to obtain the value of the specific feature energy *E*_*specific*_(*x*_*i*_).

(1) Sparse matching constraints for energy

**Fig 7:**
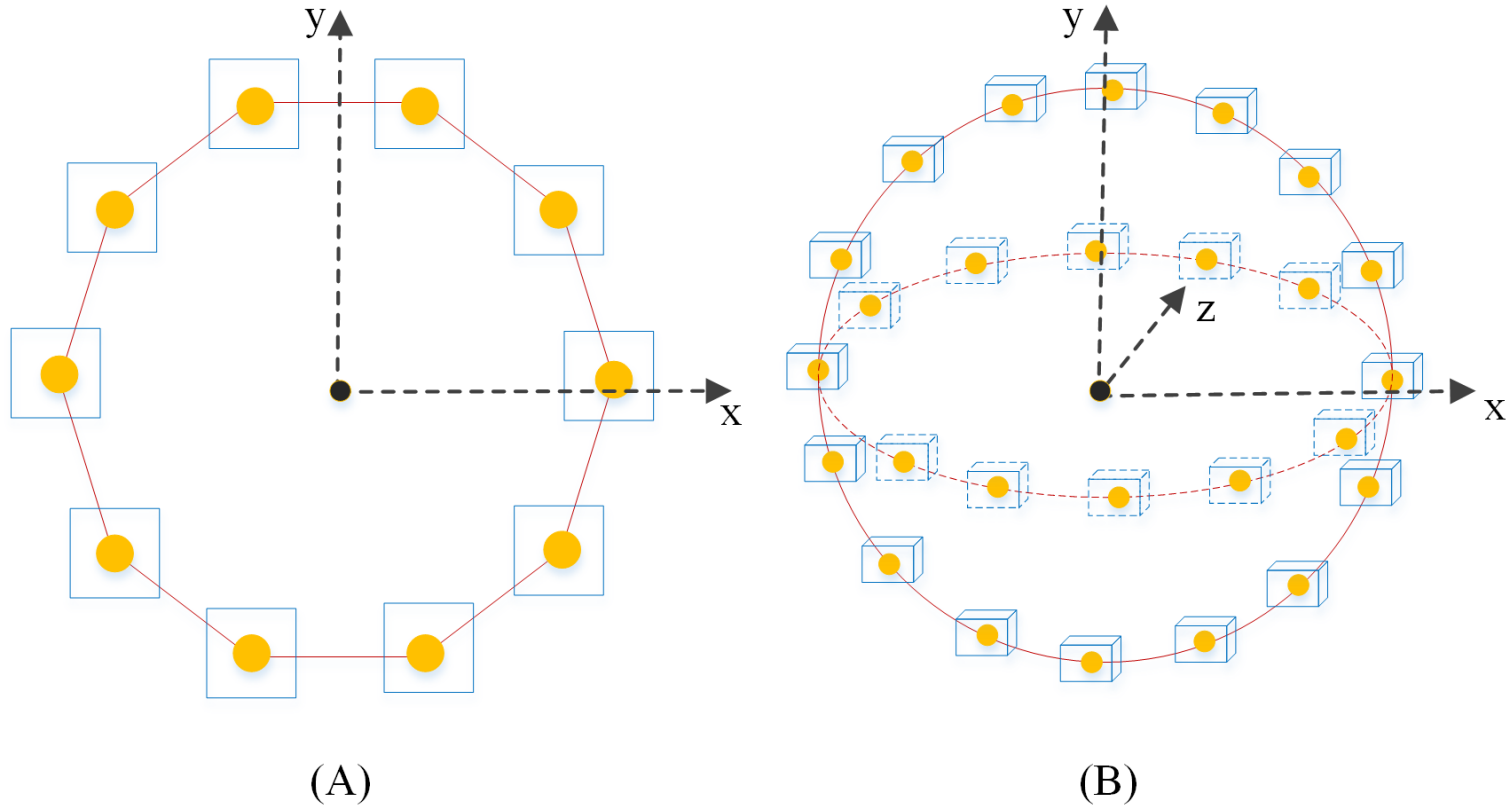
Sketch map for the selection of testing sets in the model. (A) Two-dimensional display image of the image block; and (B) three-dimensional display image.

We conduct Gabor filtering on CT images and select image blocks on the liver boundary from the Gabor images to establish the feature dictionary *D*_*gabor*_. The model updates testing sets continuously according to the vertex post of the deformable model during the deformation process. Fig 7 shows the selection methods for testing sets on the initial post of the deformable model. We select image blocks of the same size as the testing set at the vertex of the deformable model and calculate the restructuring error of each image block following every deformation according to the established dictionary and sparse coding. If the restructuring error of an image block is less than the acceptable threshold, then the intensity and boundary energies of this vertex will be set to zero. If the error is greater than the threshold, then the energy values will not be changed to allow for continuous deformation to be realized under the effect of intensity energy and boundary energy until all image blocks errors are less than the threshold and the deformation process stops.

This paper obtains the sparse coding *X*_*gabor*_ of the testing samples via the established dictionary *D*_*gabor*_. We then calculate the restructuring error of each sample based on $Y_{testing}^{i}$, the dictionary and the sparse coding.

$$L_{{}_{gabor}}^{i}={\left\|Y_{gabor}^{i}-DX_{gabor}\right\|}_{2},i=1,2,\cdots n$$ (23)

If the restructuring error of a sample is less than the threshold, then

$$L_{{}_{gabor}}^{i}\leq \sigma ,i=1,2,\cdots n$$ (24)

The energy of the corresponding vertex in this testing set will be set zero, and other vertexes will continue to deform until the restructuring errors of all testing sets are less than the threshold *σ*.

The weighting coefficient *ω*_*exrenal*_ of the energy function (10) of the original deformable model is calculated by the following formula:

$$\omega_{external}=\left\{ \begin{array}{l} 0\;s.t.\;L_{{}_{gabor}}^{i}\;\leq \sigma ,i=1,2,\cdots n\\ L_{{}_{gabor}}^{i}\;s.t.\;L_{{}_{gabor}}^{i}\;>\sigma ,i=1,2,\cdots n \end{array}\right.$$ (25)

## Experimental results and discussions

This paper conducts algorithm testing based on the CT data provided in MICCAI 2007. The data sets include 20 sets of training data, 10 sets of testing data, and the evaluation standards for segmentation results. The pixel interval of all data is 0.55–0.8 mm. The distance between two sections is 1–3 mm, and overlapping does not occur between two sections. The experiment mainly analyzes the effectiveness of sparse statistical shape models for algorithm building and discusses the intensity energy, boundary energy, and sparse matching constraints of sparse statistical shape models. The results show that the algorithm established in this paper can be used to accurately segment the liver.

**Fig 8:**
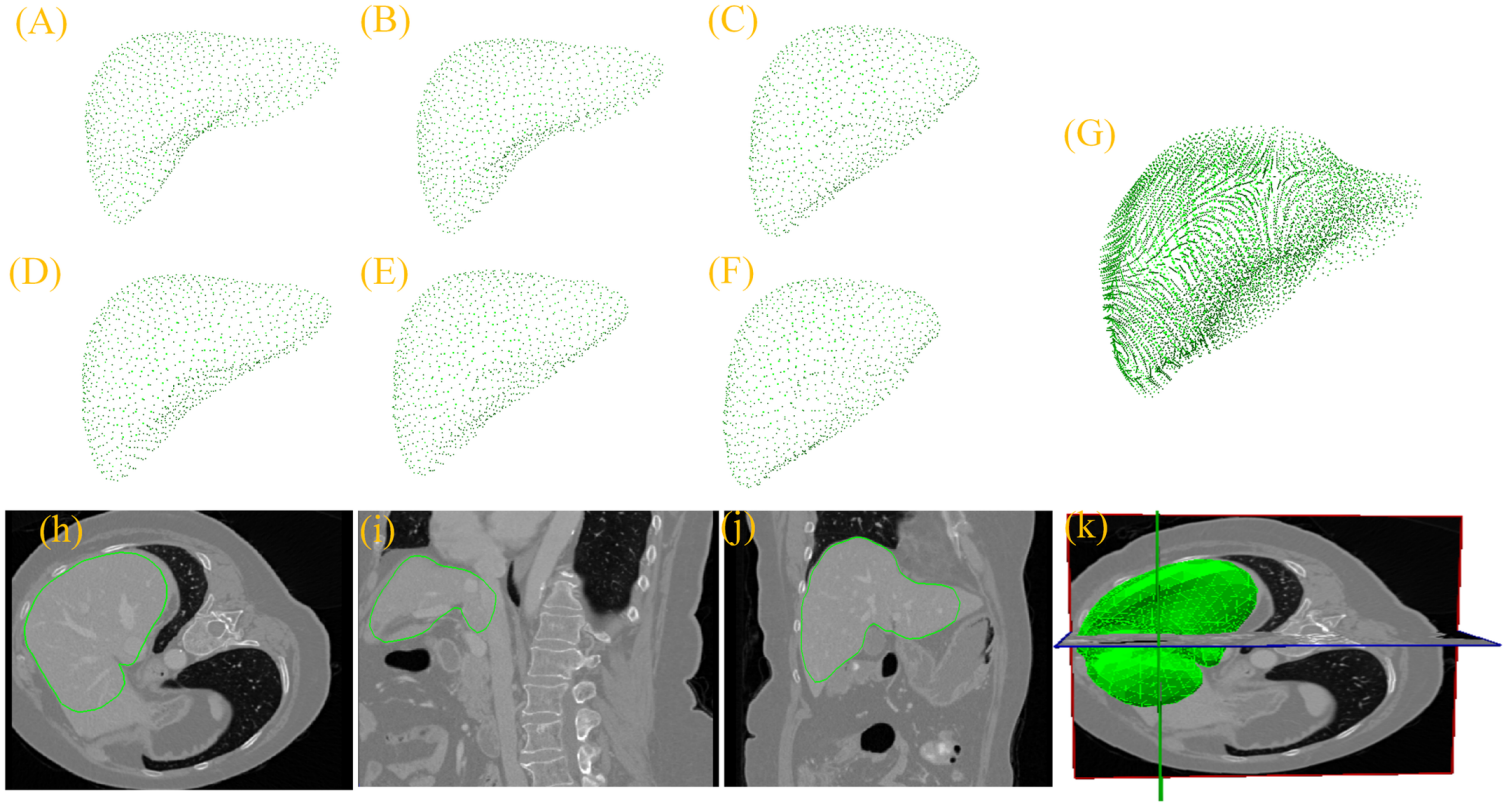
Process of building the sparse statistical shape model. (A)-(F) Six groups of a priori models; (G) overlapping result after registering the six groups of data into the same coordinate space; (H)-(J) sparse statistical shape model expressed on the cross section, vertical plane, and coronal plane of the original image; and (K) three-dimensional image of the sparse statistical shape model on the original image.

Fig 8 shows the process of building the sparse statistical shape model for the proposed algorithm. Panels (A)-(F) represent six groups of gold standard point cloud data for the liver; (G) illustrates the results of the gold standard point cloud data after registration and overlap; (H)-(J) express the sparse statistical model on the cross section, vertical plane, and coronal plane of the original image; and (K) shows the three-dimensional image of the sparse statistical shape model on the original images. As shown in Fig 8, before building of the sparse statistical shape model, the proposed method can be used to accurately register the six groups of data into the same coordinate space so that the liver shape structure and direction of each data group mutually corresponding to each other. Additionally, panels (H)-(K) indicate that the initial pose of the sparse statistical model in the original image is more accurate; thus, it has effectively reduced the errors in the traditional statistical model because of the inaccuracy of the initial pose.

**Fig 9:**
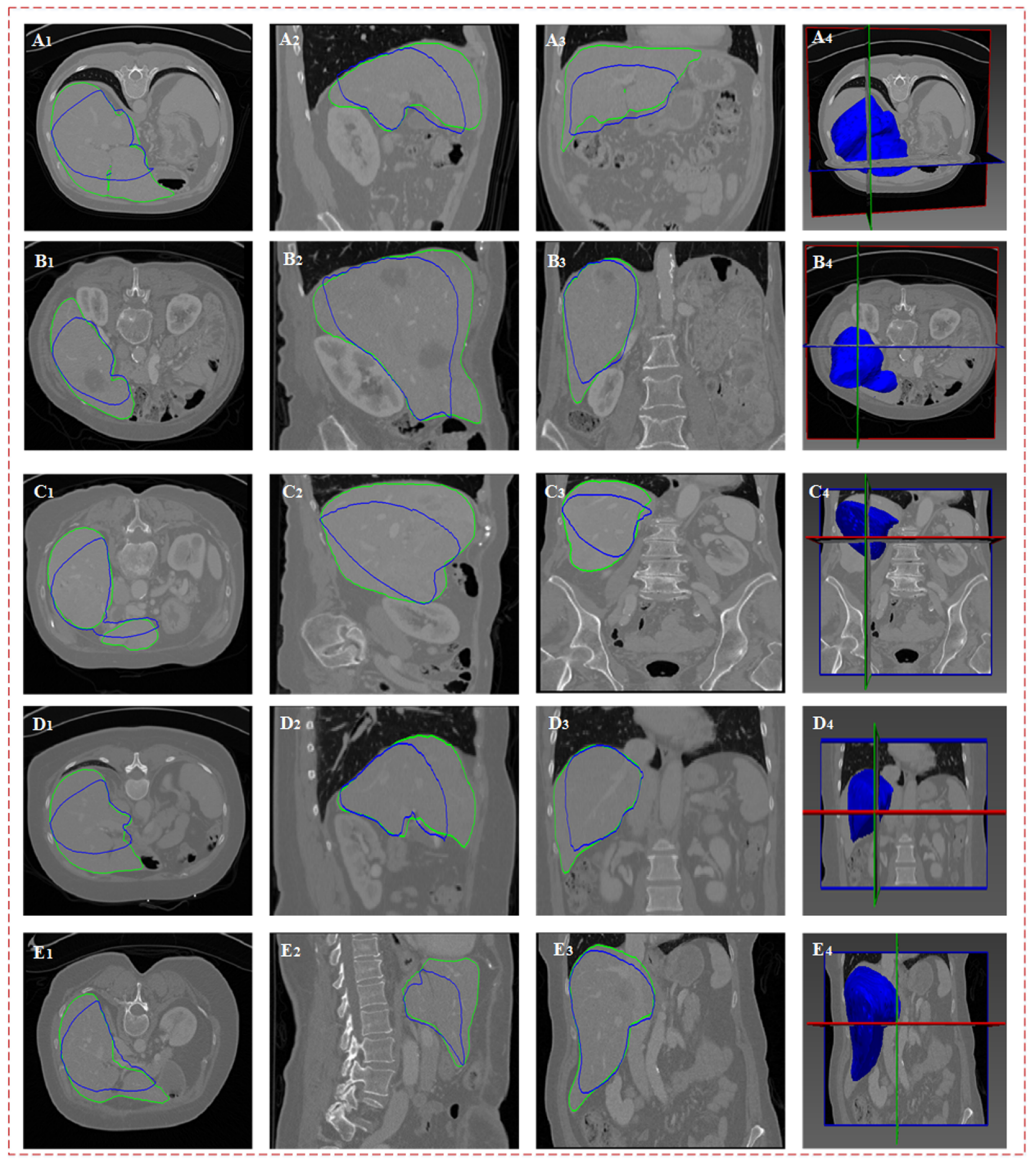
Sparse statistical shape model on the original image. The first to five rows show the five groups of original CT data; the first to fourth columns show the display results of the same group of data on the cross section, vertical plane, coronal plane, and three-dimensional space; the green line indicates the real liver boundaries of the original image; and the blue line indicates the sparse statistical shape model built based on the proposed algorithm.

Fig 9 expresses the sparse statistical model on the five original CT images. Panels (A_1_)-(A_4_), (B_1_)-(B_4_), (C_1_)-(C_4_), (D_1_)-(D_4_) and (E_1_)-(E_4_) each show the segmentation results of the five groups of data on the cross section, vertical plane, coronal plane and three-dimensional space. In this figure, the green line indicates the real liver boundaries of the original image, and the blue line indicates the sparse statistical shape model built based on the proposed algorithm. As shown in the figure, the established sparse statistical shape model locates the same coordinate space as the liver contour in the original image and matches well with the shape structure of the liver contour. Although errors occur in the selection of mark points during dictionary training and testing and the sparse statistical shape model varies significantly in local regions where the real liver boundaries are in the original image, the energy constraint model constructed in this study can drive the sparse statistical shape model to converge with the real liver boundaries.

**Fig 10:**
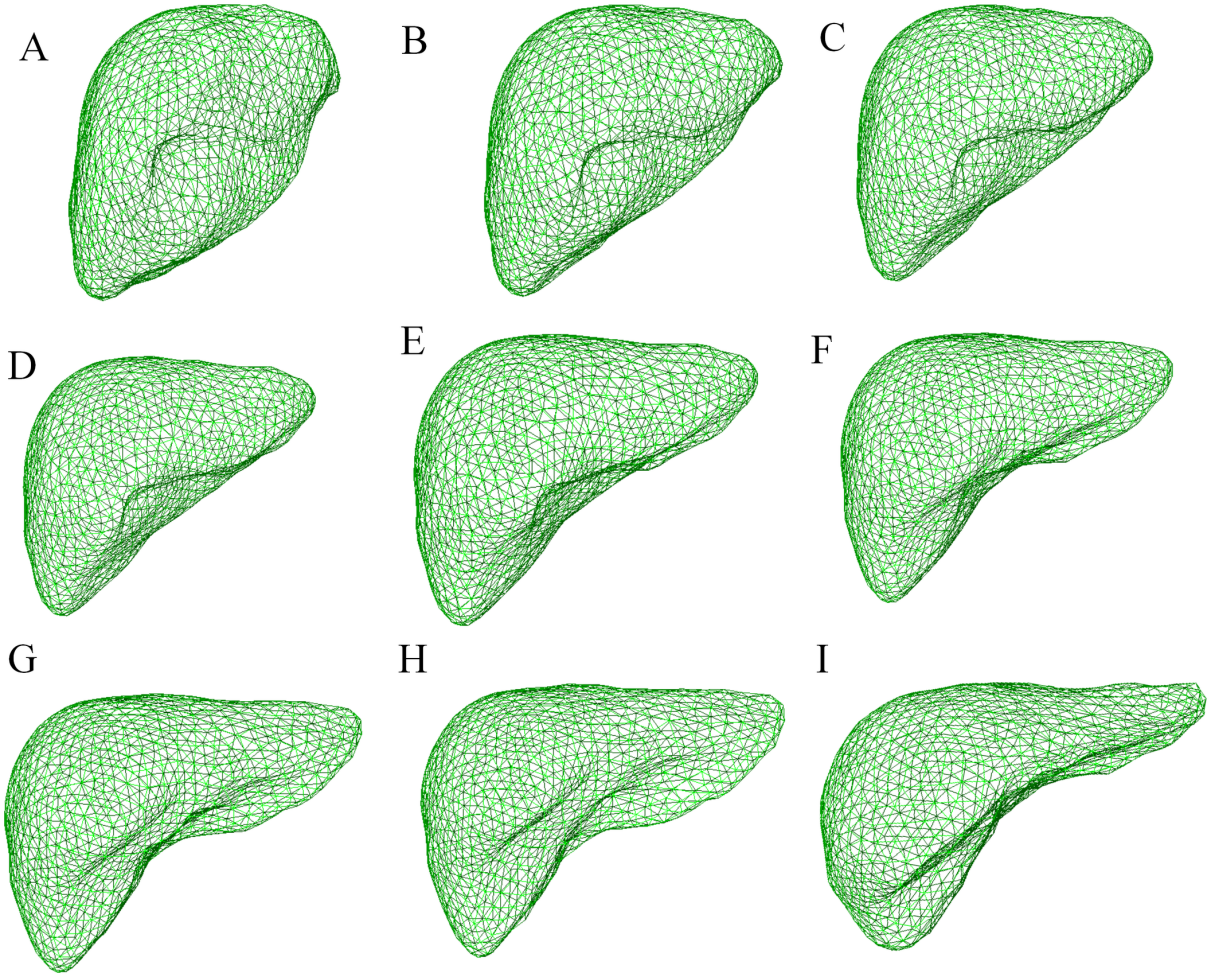
Deformation process of the sparse statistical shape model under energy constraints. (a)-(i) Results of the 1st, 3rd, 5th, 7th, 9th, 11th, 13th, 15th, and 17th iterative computations of the model.

Fig 10 shows the deformation process of the sparse statistical shape model under energy constraints. Panels (a)-(i) show the results of the 1^st^, 3^rd^, 5^th^, 7^th^, 9^th^, 11^th^, 13^th^, 15^th^, and 17^th^ iterative computations of the model. As shown in the figure, the sparse statistical shape model is gradually deformed under the constraints of the intensity and boundary energy and simultaneously ensures the smoothness of the model.

**Fig 11:**
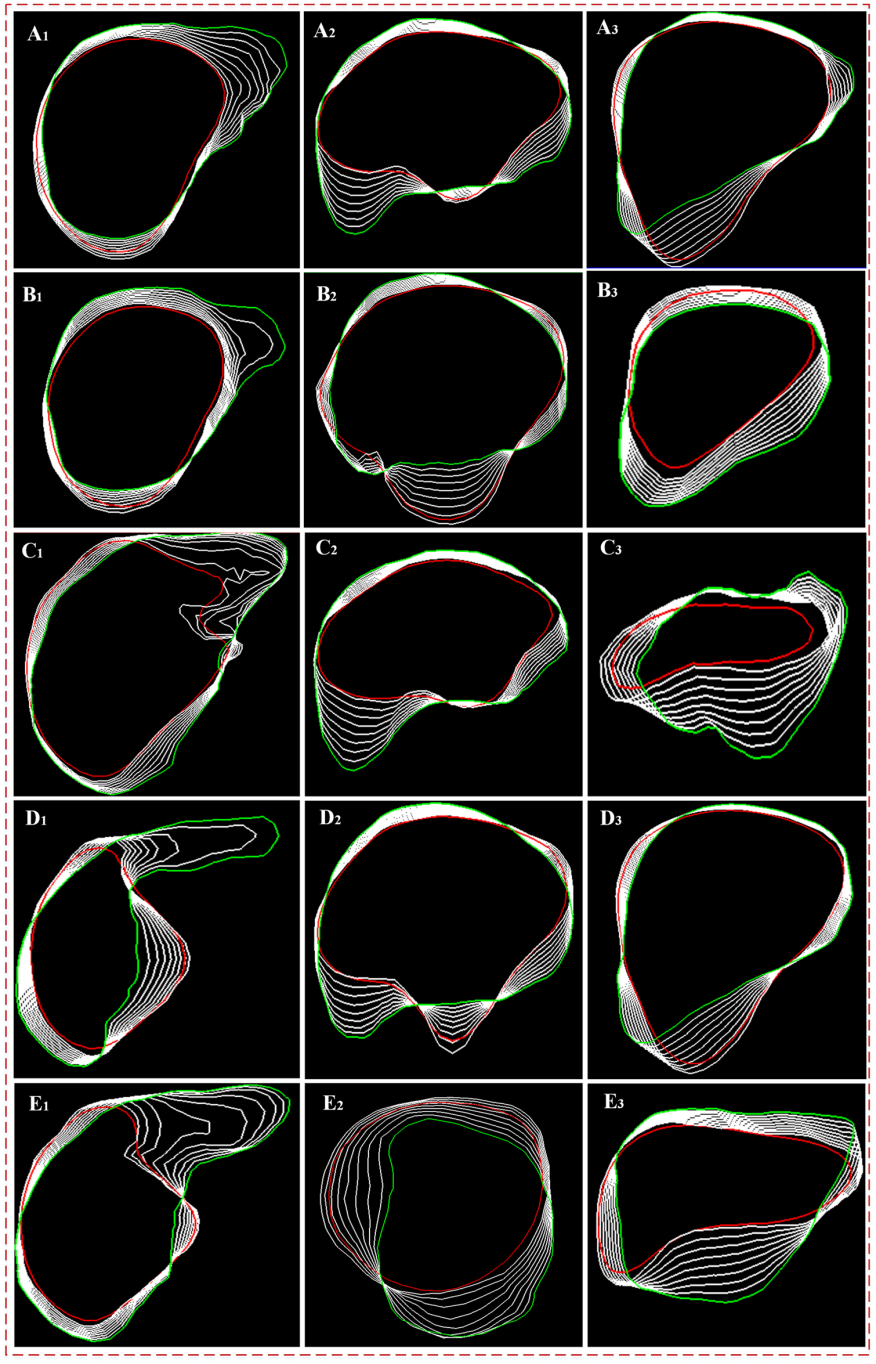
Deformation process of the sparse statistical shape model on the two-dimensional section. The first to fifth rows show the five groups of data, and the first to third columns show the cross section, vertical plane, and coronal plane during the deformation process.

Fig 11 shows the deformation process of the sparse statistical shape model on the two-dimensional section. (A_1_)-(A_3_), (B_1_)-(B_3_), (C_1_)-(C_3_), (D_1_)-(D_3_) and (E_1_)-(E_3_) show the results of the five deformable models on the cross section, vertical plane, and coronal plane. In this figure, the red line indicates the initial sparse statistical shape model, the green line indicates the gold standard liver boundaries, and the white line indicates the intersections of the model with different sections during the deformation process. As shown in the figure, the initial sparse statistical shape model, which is driven jointly by the intensity energy, boundary energy, and sparse matching constraints, can gradually deform to obtain a shape that is close to the actual liver boundaries. At the early stage, the deformation extent of the model is small. Then, as the model boundaries approach the liver boundaries and the sparse matching constraints decline, the deformation extent of the model decreases significantly. On the smooth liver surface, because of the high matching degree between image blocks and dictionaries in the building process of the sparse statistical shape model, the sparse matching constraints are relatively small, thereby decreasing the deformation extent of the model. However, in the grooved regions and sharp corners representing detailed information, the low matching degree between image blocks and dictionaries leads to relatively large sparse matching constraints, which increases the deformation extent of the model. As a result, the intensity energy, boundary energy, and sparse matching constraint model can effectively drive the statistical shape model to represent the sharp corners of the liver in the original image.

**Fig 12:**
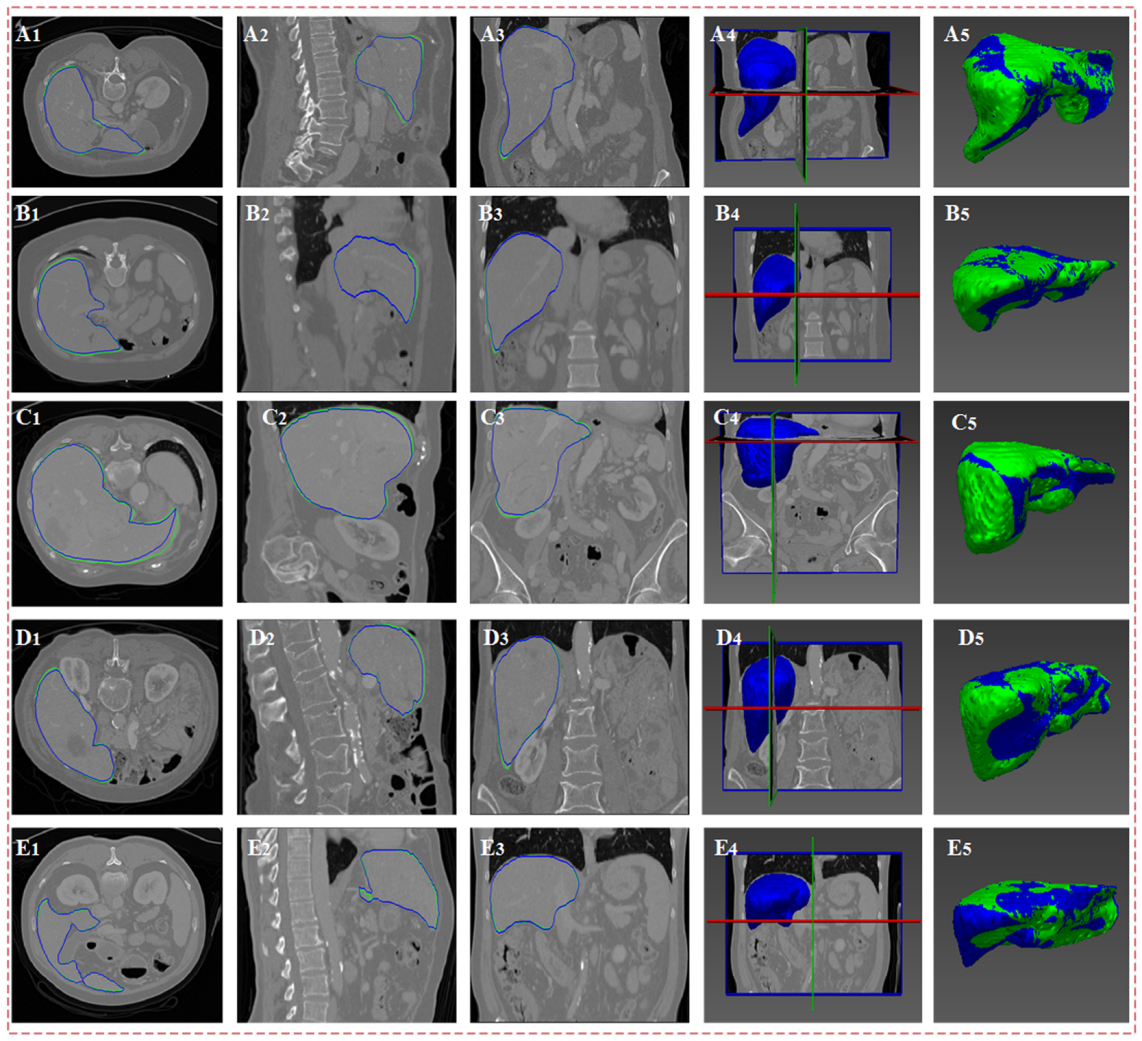
Deformation results. The first to fifth rows show the five groups of CT data. (A_1_)-(A_4_), (B_1_)-(B_4_), (C_1_)-(C_4_), (D_1_)-(D_4_) and (E_1_-E_4_) show the deformation results of the five groups of data on the cross section, vertical plane, coronal plane and three-dimensional space. (A_5_), (B_5_), (C_5_) and (D_5_) show the overlapping images of the deformation results of the five groups of data with the real liver boundaries. The green line indicates the real liver boundaries of the original image, and the blue line indicates the segmentation result based on the proposed algorithm.

In Fig 12, panels (A_1_)-(A_4_), (B_1_)-(B_4_), (C_1_)-(C_4_), (D_1_)-(D_4_) and (E_1_-E_4_) express the deformation results of the five sparse statistical shape models that are driven by the intensity energy, boundary energy and sparse matching constraints on cross section, vertical plane, coronal plane and three-dimensional space. In this figure, the green line indicates the real liver boundaries in the original image, the blue line indicates the deformation results, and the blue shape area shows the three-dimensional distribution of the deformation results and the pose of the deformation results in the original CT image. Panel (A_5_), (B_5_), (C_5_), (D_5_) and (E_5_) represent the overlapping images of the deformation results of the five groups of data and the real liver boundaries. As shown in the figure, the proposed algorithm can be used to segment the liver with high accuracy.

**Fig 13:**
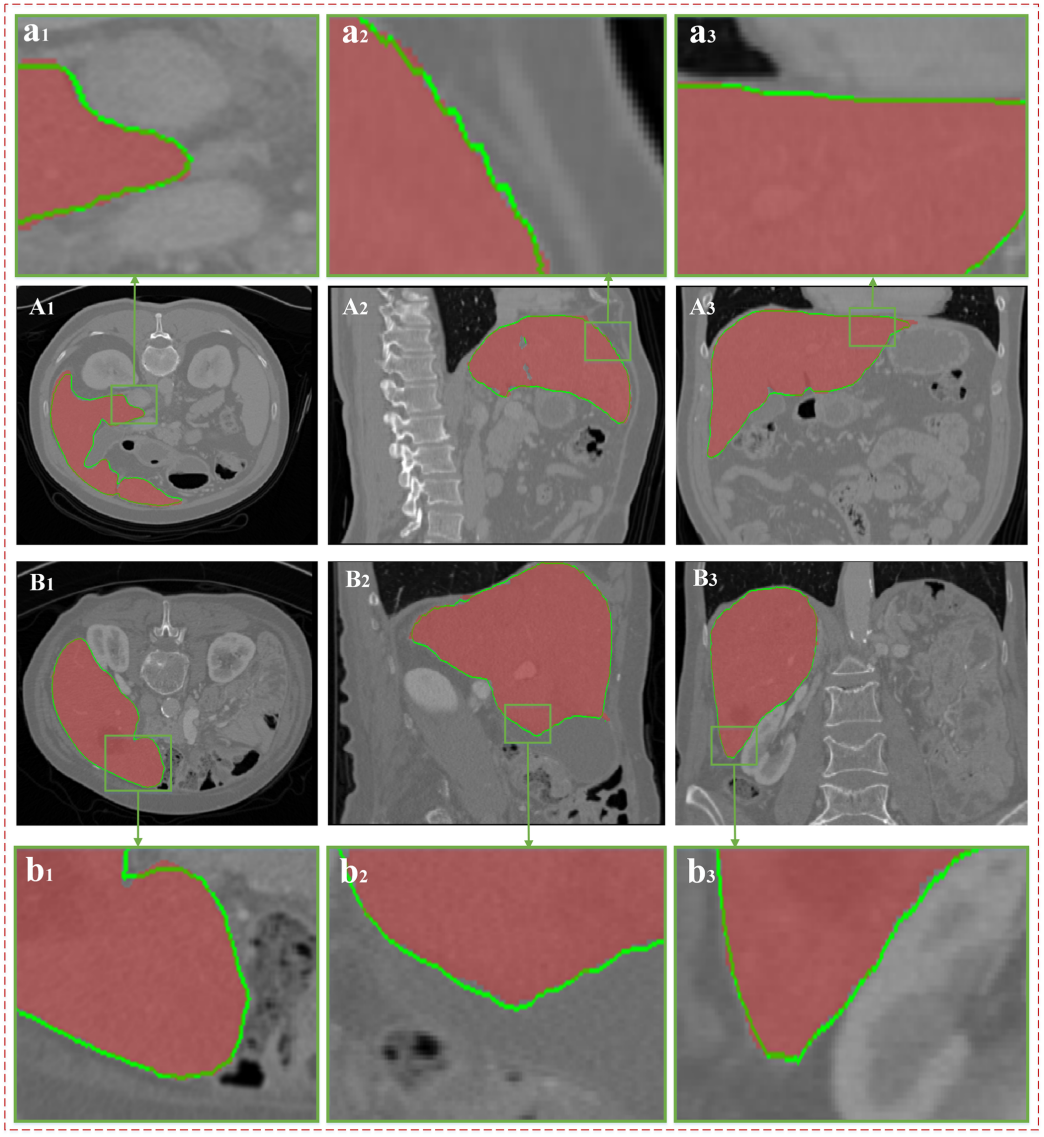
Segmentation results. (A) and (B) show the two groups of CT data; (A1), (A2) and (A3) express the same groups of data on the cross section, vertical plane, and coronal plane, respectively; and (a_1_), (a_2_) and (a_3_) show the partially enlarged images corresponding to the green areas in (A_1_), (A_2_) and (A_3_), respectively. The red area in every image indicates the real liver boundaries, and the green line indicates the liver segmentation results based on the proposed algorithm.

Fig 13 shows the liver segmentation results of two groups of CT data based on the proposed algorithm. Panels (A_1_)-(A_3_) and (B_1_)-(B_3_) show the segmentation results of the two groups of data on cross section, vertical plane, and coronal plane. The red area indicates the real liver boundaries, and the green line indicates the liver segmentation results based on the proposed algorithm. Panels (a_1_)-(a_3_) and (b_1_)-(b_3_) show the partially enlarged images corresponding to the green areas in (A_1_)-(A_3_) and (B_1_)-(B_3_), respectively. As shown in the figure, when the boundary intensity information is not obvious or burrs occur around the liver boundaries, minor errors may occur in the liver segmentation results based on the proposed algorithm. In general, the difference between the segmentation results and the gold standard data is small.

## Analysis of segmentation results

Table 1 provides the testing results regarding the segmentation accuracy of the algorithm in this paper based on five sets of randomly selected data. The table shows that the mean values of the volumetric overlap error (VOE), relative volume difference (RVD), average symmetric surface distance (ASSD), root mean square symmetric surface distance (RMSSSD), and maximum symmetric surface distance (MSSD) reached 4.8±0.7%, 1.8±0.4%, 0.8±0.1 mm, 1.4±0.4 mm and 15.9±4.3 mm, respectively, and the average scores are 82±3.5, 88±2.6, 81±1.3, 78±9.0 and 82±4.5, respectively. These results show that the algorithm in this paper can make the sparse statistical shape model effectively converge to the liver boundaries under the joint effect of intensity energy, boundary energy, and sparse matching constraints, thereby obtaining more accurate segmentation results.

**Table 1:** Analysis of the segmentation results errors of the proposed algorithm.

Data	VOE		RVD		ASSD		RMSSSD		MSSD		Total
	[%]	Score	[%]	Score	[mm]	Score	[mm]	Score	[mm]	Score	Score
1	3.7	88	1.8	89	0.9	80	1.9	70	14.8	83	82
2	5.3	80	1.5	91	0.8	81	1.2	83	11.2	88	85
3	4.9	83	2.1	86	0.6	83	0.8	89	18.4	80	84
4	5.4	79	2.3	84	0.9	80	1.3	82	13.1	84	82
5	5.0	81	1.4	92	0.7	82	1.7	68	21.9	76	80
AVG	4.8±0.7	82±3.5	1.8±0.4	88±2.6	0.8±0.1	81±1.3	1.4±0.4	78±9.0	15.9±4.3	82±4.5	82±1.9

Table 2 presents comparisons of the segmentation results errors of the algorithm in this paper as well as six other algorithms [24–30], which included algorithms that presented better segmentation results from the MICCAI competition and those used for experimental analyses based on MICCAI data and evaluation methods in recent years. The table shows that the segmentation accuracy of the algorithm in this paper is higher than that of other algorithms, and the total score based on five segmentation result evaluation standards reached 82, which is far higher than the total scores of 69, 67, 64, 62, 60 and 53 achieved by the other algorithms. The ASSD of the segmentation accuracy of the algorithm in this paper was reduced by 0.6 mm, 0.6 mm, 0.7 mm, 0.9 mm, 1.0 mm and 1.4 mm compared with those of the other algorithms; the average VOE was reduced by 2.5%, 2.9%, 4.1%, 4.3%, 5.6% and 7.7%; the RMSSSD error was reduced by 1.7 mm, 1.8 mm, 2.0 mm, 1.9 mm, 1.8 mm and 3.0 mm; and the MSSD was reduced by 10.9 mm, 14.2 mm, 13.4 mm, 14.9 mm, 9.3 mm and 16.5 mm. These result show that the three algorithms in this paper can enhance the effectiveness and accuracy of liver segmentation.

**Table 2:** Error comparisons among other segmentation methods.

Methods	VOE		RVD		ASSD		RMSSSD		MSSD		Total
	[%]	Score	[%]	Score	[mm]	Score	[mm]	Score	[mm]	Score	Score
**SP-SSM**	**4.8±0.7**	**82**	**1.8±0.4**	**88**	**0.8±0.1**	**81**	**1.4±0.4**	**78**	**15.9±4.3**	**82**	**82**
AMEM[^30^]	6.5±0.6	75	2.8±0.9	85	1.1±0.3	74	2.2±0.8	69	22.8±7.6	70	74
Oliveira[^24^]	7.3	71	2.2	82	1.4	66	3.1	58	26.8	65	69
Heimann[^25^]	7.7±1.9	70	1.7±3.2	88	1.4±0.4	65	3.2±1.3	55	30.1±10.2	60	67
Saddi[^26^]	8.9±1.8	65	1.2±4.4	80	1.5±0.4	62	3.4±0.8	52	29.3±8.4	62	64
Chi[^27^]	9.1±2.8	65	2.6±6.3	73	1.7±0.6	58	3.3±1.2	54	30.8±9.2	60	62
Seghers[^28^]	10.4±2.5	58	6.8±2.3	64	1.8±0.4	55	3.2±1.1	56	25.2±10.1	67	60
Rikxoort[^29^]	12.5±1.8	51	1.8±4.2	80	2.4±0.3	40	4.4±1.5	40	32.4±13.7	57	53

Table 3 shows the average time statistics for the MICCAI data segmentation experiment using the methods proposed in this paper on a computer with a CPU frequency of 3.4 GHz. The Oliveira [24] method in Table 1 does not provide the segmentation time from the related literature; thus, only the other five methods are compared in this chapter. The table shows that although the computational efficiency of the SP-SSM algorithm proposed in this paper is improved compared with the other types of algorithms, it was reduced by 8.8 min relative to the same type of Chi [27] and Seghers [28] algorithms. The results show that the construction of a liver sparse statistical shape model based on the sparse coding theory in the SP-SSM algorithm is highly efficient, and they also indicate the effectiveness of the gray energy, the boundary energy and the sparse matching energy constraint method.

**Table 3:** Comparison of segmentation performance.

Parameters	SP-SSM	AMEM [30]	Heimann [25]	Saddi [26]	Chi [27]	Seghers [28]	Rikxoort [29]
Type	Statistical	Deformation	Deformation	Deformation	Statistical	Statistical	Learning
Segmentation time	21.2 min	4.9 min	7 min	5.5 min	30 min	30 min	45 min
Computer performance	3.4 GHz	3.4 GHz	3 GHz	2 GHz	3 GHz	3.4 GHz	3.2 GHz

## Conclusions

Considering the complexity of CT images for the liver, this study creates dictionaries for a normalized a priori shape model and the mark points. Then, the sparse coefficient is calculated based on the mark point dictionary and the sparse statistical shape model is built based on the a priori shape model dictionary. Subsequently, the specific boundaries of the original image are obtained by using the pose of the sparse statistical model in the original image so that the intensity energy and boundary energy are built. Finally, a sparse matching constraint model is established based on the liver boundary information dictionary and the Gabor information of the original image. Accordingly, the deformation scope and extent of the sparse statistical shape model can be controlled effectively and the model can closely approximate the liver boundaries, thereby realizing accurate liver segmentation.

In experimental section, this study utilized public liver data sets provided by MICCAI 2007 for the experimental analysis. According to the experimental results, the sparse statistical shape model built based on the proposed algorithm can represent the initial liver boundaries in the original image and approach the real liver boundaries under the driving force of the intensity energy, boundary energy, and sparse matching constraints, thereby obtaining accurate segmentation results.

Finally, this study compares the segmentation results of the proposed model with those obtained based on the six prevailing algorithms. According to the comparison results, the proposed algorithm can more accurately extract the liver contour from the CT image. Moreover, the mean values of VOE, RVD, ASSD, RMSSSD, and MSSD are 4.8±0.7%, 1.8±0.4%, 0.8±0.1 mm, 1.4±0.4 mm and 15.9±4.3 mm, respectively.

Nevertheless, when using the proposed algorithm, researchers must manually select mark points on the liver boundaries. Although sparse coding can be applied to effectively remove the inaccurate mark points or liver models, subjective factors may still be present. In addition, in the proposed method, sparse matching constraints are implemented in the deformation process. Therefore, matching must be conducted between the image blocks corresponding to each vertex in the model and the dictionary each time an iteration begins, although this process decreases the segmentation efficiency of the proposed algorithm to some extent. Currently, the average time required for liver segmentation is approximately 21.2 minutes, which can still be significantly improved. Hence, future studies should focus on graphics processing unit-based acceleration and fully automated solutions for the proposed method.
